# Potential Risks and Spatial Variation of Heavy Metals in Water and Surface Sediment of Pattani Bay, Thailand

**DOI:** 10.3390/toxics13060477

**Published:** 2025-06-05

**Authors:** Kanjana Imsilp, Pattanasuda Sirinupong, Pun Yeesin, Wachiryah Thong-asa, Phanwimol Tanhan

**Affiliations:** 1 Department of Pharmacology, Faculty of Veterinary Medicine, Kasetsart University, Bangkok 10900, Thailand; fvetkni@ku.ac.th; 2 Department of Science, Faculty of Science and Technology, Prince of Songkla University, Pattani Campus, Pattani 94000, Thailand; pattanasuda.c@psu.ac.th; 3 Department of Technology and Industry, Faculty of Science and Technology, Prince of Songkla University, Pattani Campus, Pattani 94000, Thailand; punyeesin@gmail.com; 4 Animal Toxicology and Physiology Specialty Research Unit, Department of Zoology, Faculty of Science, Kasetsart University, Bangkok 10900, Thailand; fsciwyth@ku.ac.th

**Keywords:** heavy metals, sediments, ecological health risk

## Abstract

This investigation examined the physicochemical characteristics and heavy metal contamination within the surface sediments and aquatic environments of Pattani Bay, Thailand, throughout both wet and dry seasons. The sediments were primarily composed of fine-grained materials, specifically silt and clay, and exhibited greater propensity to absorb heavy metals from water. Notably elevated concentrations of Cd and Pb were detected, particularly within riverine sediment deposits. This indicates that riverine inputs are significant pathways of the contamination and potentially associated with historical mining activities. Seasonal fluctuations affected physicochemical parameters as well as metal concentrations. The heightened levels of Cd and Pb during the wet season were attributed to runoff phenomena. Pollution indices including the Contamination Factor (CF), pollution load index (PLI), and geoaccumulation index (Igeo) demonstrated moderate to extremely high contamination levels of Cd and Pb in certain areas. The Principal Component Analysis (PCA) suggested possible similar sources for multiple metals including Cd, Cu, Pb, and Zn. The results showed that the heavy metal pollution present is serious, especially for Cd and Pb. These could lead to high ecological health risks and so it is necessary to focus on implementing environmental management strategies for Pattani Bay.

## 1. Introduction

The urgent global environmental challenge of heavy metal contamination in water and sediment must be addressed. Marine sediments serve as the ultimate repository for heavy metals discharged into marine environments. They, thus, are the principal reservoir for these contaminants within aquatic ecosystems, which can pose substantial threats to marine biodiversity [1,2,3,4,5,6,7]. The heavy metal pollution of water bodies also generates environmental challenges and can detrimentally impact marine organisms as well [8]. The quantifications of heavy metal concentrations in marine sediments and water are, thus, crucial for monitoring and estimating environmental crises. Consequently, it is very important that the concentrations and origins of these heavy metals are elucidated and can later be managed to secure the marine ecosystems [1,9].

Bay systems represent critical land–sea interfaces. They function as pivotal transition zones for the transfer of terrigenous pollutants to marine environments. They, therefore, are optimal sites for the investigation of dispersed heavy metals. The intricate hydrodynamic conditions in an individual bay significantly influence the dynamics of transport. Sediment deposition within these environments also plays a crucial role in sustaining fine-grained particles [10,11]. These altogether could ultimately result in alterations in the spatial distribution of heavy metals within the sediments.

**Figure 1:**
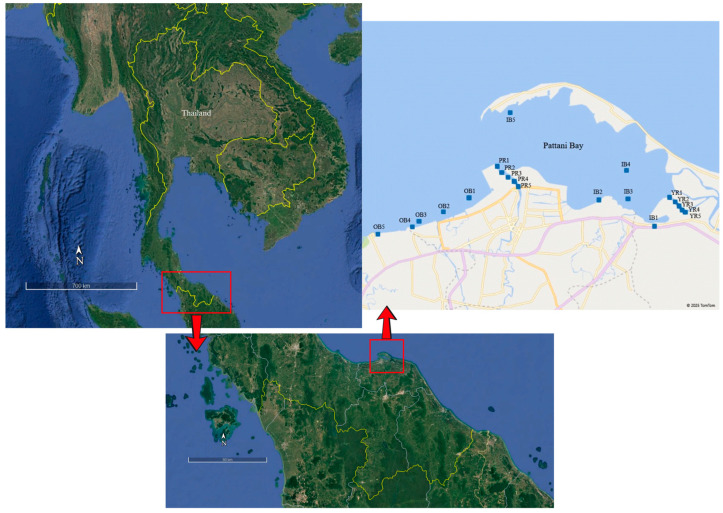
Sampling locations at in- and off-Pattani Bay (IB and OB), Yaring River (YR), and Pattani River (RR), Thailand. Map data: Google, @2020/Astrium, Maxar Technologies.

Pattani Bay (coordinates 6°51′–6°57′ N, 101°–101°21′ E) constitutes a semi-enclosed maritime feature located in the Pattani province of Southern Thailand (Figure 1). This bay serves as a center for Thailand aquaculture with an area of 74 square kilometers and an average depth of 1.1 m. The Yaring and Pattani rivers are discharged into the bay. The Pattani River basin has encountered considerable heavy metal contamination of soil and water resources over the last five decades [12]. Notable sources of this pollution have been attributed to aquaculture operations, mining endeavors, urban influence, as well as industrial waste discharges. The tin mining activities in the upper basin of Yala province have also resulted in lead (Pb) contamination in both sediment and water bodies [12,13,14]. The effluents transported via rivers may also exacerbate pollution concentrations in the aquatic environment and sediments of the bay. Elevated Pb concentrations were identified within the sediments, water, and marine flora of the Pattani River in 1986 [15]. Regular dredging operations are necessary to ensure large vessel access to Pattani Bay. These, however, can induce the mobilization of heavy metals deposited in the Pattani Bay sediment. Operations including farming, industry, mining, urban area sewage, and agricultural wastewaters have significantly contributed to the pollution levels of multiple toxicants along with heavy metals [12]. The potential ecological risks of heavy metal contamination in Pattani Bay can be derived from the influence of these heavy metals originating from both natural and anthropogenic activities on riverine and marine sediments. Sediments commonly serve as a dynamic matrix for various biological and chemical processes. They also provide nutritional resources for marine biota. Their excessive contamination would cause health risks for both humans and other organisms. These also allow various toxicants to infiltrate the food web and impact marine biota in this area and others [14,16,17,18,19,20].

A variety of environmental indices are employed to quantify pollution levels in aquatic ecosystems. These indices include the contamination degree (CD), modified degree of contamination (mCD), contamination factor (CF), geoaccumulation index (Igeo), pollution load index (PLI), potential ecological risk index (RI), and Sediment Quality Guidelines (SQGs) [21,22,23,24,25,26]. A previous investigation did not evaluate the environmental quality of Pattani Bay [27]. The distribution and enrichment status of heavy metals within Pattani Bay also remained inadequately characterized prior to this study. It is also necessary to investigate and assess the environmental quality of Pattani Bay after the 2020 dredging operations. This could provide useful references for governmental agencies. This study, therefore, aimed to determine the pollution status of heavy metals in the sediments of Pattani Bay and elucidate the relationship between heavy metals and various physicochemical parameters including sediment texture and total organic carbon (TOC). Investigation of the origins of heavy metals in sediments and the ecological risk profiles within Pattani Bay is vital for comprehending the ramifications of anthropogenic activities on the ecological landscape.

The objectives for this study were to (1) investigate the concentrations and distribution patterns of heavy metals (Cd, Co, Cr, Cu, Fe, Mn, Ni, Pb, and Zn) in the sediments of Pattani Bay; (2) evaluate the potential ecological risks associated with heavy metal pollution; and (3) investigate the physicochemical factors of the sediment that influence heavy metal distribution. The outcomes retrieved from this study are expected to establish a foundational scientific framework for the ecological preservation and pollution management of Pattani Bay.

## 2. Materials and Methods

### 2.1. Description of the Study Area and Sample Collection

This research was conducted at Pattani Bay, Pattani province, Thailand. The Pattani Bay receives water flow from two principal rivers, the Yaring River and the Pattani River. The sampling sites were categorized into the in-bay and off-bay locations. Sample collections were performed in the wet and dry seasons at 20 distinct stations (Figure 1). These stations included in Pattani Bay (IB1–5), off Pattani Bay (OB1–5), Yaring River (YR1–5), and Pattani River (PR1–5). Surface sediment (1 kg) at a depth of 1–15 cm and water (1 L) were collected using stainless steel materials. All samples were subsequently placed in polyethylene containers and refrigerated. The surface sediment samples underwent oven-drying at 70 °C and were then sieved through a 2 mm nylon sieve to eliminate larger debris. The water samples were filtered using No. 4 Whatman^®^ filter paper prior to heavy metal analysis. All samples were preserved and prepared for heavy metal analysis according to the standard of the American Public Health Association (APHA, AWWA [28]).

All samples were oven-dried at 105 °C for 24 h and subsequently weighed for grain size analysis of surface sediments [29]. They were differentiated by the wet method using a vibration sieve set. Each individual residual-containing sieve was oven-dried under the same conditions and various sediment fractions were weighed [30]. Organic matter demonstrates its affinity for heavy metals within the aquatic milieu via mechanisms of adsorption and complexation [31]. The pH of surface sediments and electrical conductivity (EC) were assessed employing a pH meter and a soil and solution electrical conductivity meter, respectively. The organic carbon content was quantified through the exothermic oxidation of organic matter using H_2_SO_4_, followed by titration with Fe(NH_4_)_2_(SO_4_)_2_·6H_2_O [32].

### 2.2. Heavy Metal Analysis

The acid digestion procedure for nine heavy metals (Cd, Co, Cr, Cu, Fe, Mn, Ni, Pb, and Zn) was performed according to the standardized protocols of the APHA [28]. Sediment samples underwent drying in an oven until they attained a stable mass. After cooling, the sediments were homogenized and pulverized into fine particles to eliminate larger aggregates. An aliquot (0.5 g) of individual dried sediment sample was meticulously weighed in triplicate and subsequently digested with high-purity 69% nitric acid on a hot plate set at 120 °C. The process was performed until the brown vapor disappeared and the solution became pale yellow and transparent. When the digestion process was complete and the solution cooled down, it was filtered using No. 4 Whatman^®^ filter paper. The filtrate was subsequently diluted to a final volume of 25 mL with ultrapure water (18.2 MΩ-cm Milli-Q Water) for heavy metal analysis by the Flame Atomic Absorption Spectrophotometry (FAAS).

The FAAS (SpectrAA 240B, Agilent Technologies, Victoria, Australia) was equipped with a deuterium background corrector. The analyses of all heavy metals were conducted in absorbance mode at the optimal wavelengths: Cd at 228.8 nm, Co at 240.7 nm, Cr at 357.9 nm, Cu at 324.7 nm, Fe at 248.3 nm, Mn at 279.5 nm, Ni at 232.0 nm, Pb at 217.0 nm, and Zn at 213.9 nm. The atomization process was conducted in an air/acetylene flame with flow rates of 13.3–2.9 L/min for Cr and 13.5–2.0 L/min for the remaining elements. The hollow cathode lamp currents were calibrated to 4.0 mA for Cd, Cu, and Ni; 5.0 mA for Fe, Mn, Pb, and Zn; and 7.0 mA for Co and Cr. The detection limits of each heavy metal were Cd 0.002 mg/L, Co 0.02 mg/L, Cr 0.01 mg/L, Cu 0.008 mg/L, Fe 0.02 mg/L, Mn 0.005 mg/L, Ni 0.02 mg/L, Pb 0.05 mg/L, and Zn 0.002 mg/L.

### 2.3. Quality Control and Assurance

The quantification of each metal was derived from its respective calibration curve. Calibration curves for all heavy metals, exhibiting an *r*^2^ value of 0.999, were suitable for metal concentration calculations. Each heavy metal concentration in the samples was reported as the mean of three separate measurements.

The standard reference (CRM277) was employed to ensure precision and integrity of heavy metal analysis. The precision and accuracy of heavy metal analyses were retrieved through five replicates. The recovery rates for spiked samples of investigated heavy metals ranged from 95% to 107%. The accuracy of the analyses conducted on repeated samples was maintained within ±10%, and the discrepancies between the measured values and the verified values were less than 5%.

### 2.4. The Environmental Assessment Indices

The indices of contamination were used for the evaluation of surface sediment and aqueous environments in this investigation including the heavy metal evaluation index (HEI), contamination factor (CF), pollution load index (PLI), contamination degree (CD), enrichment factor (EF), and geoaccumulation index (Igeo). The details of specific indices are described below.

#### 2.4.1. Heavy Metal Evaluation Index (HEI)

The heavy metal evaluation index (HEI) is a parameter used to evaluate the combined influences of all heavy metals on water quality. It is calculated using the following equation Tamasi and Cini [33]:

(1) $$\mathrm{HEI}=\sum_{i=1}^{i=n}\frac{C_{i}}{{\mathrm{MAC}}_{i}}$$

where C_i_ is the concentration of i heavy metal in water (mg/L), and MAC_i_ is the highest standard permissible level of i heavy metal, adapted from the drinking water quality standards of the WHO [34]. The classification of HEI is as follows: low level of contamination (HEI < 40), medium level of contamination (40 ≤ HEI < 80), and high level of contamination (HEI ≥ 80). These pose a significant risk to water quality and potentially to human health [35].

#### 2.4.2. Contamination Factor (CF)

The contamination factor (CF) is used to determine the contamination state of the sediment. It is calculated using the following equation Hakanson [36]:

(2) $$\mathrm{CF}=\frac{C_{\mathrm{metal}}}{C_{\mathrm{background}}}$$

where C_metal_ is the content of heavy metal in the sediment, C_background_ is the natural background value of that metal [37]. The Taylor’s reference background and regional background concentrations from China for Cd, Co, Cr, Cu, Fe, Mn, Ni, Pb, and Zn were used and were 0.13 mg/kg, 5.58 mg/kg, 78.03 mg/kg, 25 mg/kg, 35,000 mg/kg, 644 mg/kg, 29.47 mg/kg, 23.88 mg/kg, and 69.88 mg/kg, respectively [38,39,40]. The contamination levels by CF are categorized into 4 classes; low contamination (CF < 1), moderate contamination (1 ≤ CF < 3), considerable contamination (3 ≤ CF < 6) and very high contamination (CF > 6).

#### 2.4.3. Pollution Load Index (PLI)

The pollution load index (PLI) is calculated by obtaining the *n*th root of the *n* number of multiplied contamination factor (CF) values. The PLI of an individual sediment site is obtained as a contamination factor (CF) of each metal and is assessed using the following equation:

(3) $$\mathrm{PLI}=\sqrt[{n}]{{\mathrm{CF}}_{1}\times {\mathrm{CF}}_{2}\times \ldots \times {\mathrm{CF}}_{n}}$$

The pollution loads by PLI are categorized into 3 classes; no pollution (PLI < 1), baseline levels (PLI = 1), and polluted (PLI > 1) [41,42].

#### 2.4.4. Contamination Degree (CD)

The contamination degree (CD) represents the average concentration of a specific heavy metal in contaminated sediment relative to the pre-industrial “background or baseline value” of that particular metal [43]. It includes the cumulative total of all contamination factors (CFs), thereby reflecting the comprehensive level of contamination present in surface sediments at a specific location. The classification of the CD is as follows: CD < 6 signifies low degree of contamination, 6 ≤ CD <12 denotes moderate degree of contamination, 12 ≤ CD < 24 indicates significant degree of contamination, and CD ≥ 24 states very high degree of contamination [43].

#### 2.4.5. Enrichment Factor (EF)

The enrichment factor (EF) is a useful tool for estimating and monitoring anthropogenic heavy metal pollution over time [21,24,44,45]. The Fe metal was used as a normalizer to calculate EF. It shows the difference in sediment granular Fe, which exhibited a good correlation with all heavy metals [46]. The EF is calculated using the following equation [47]:

(4) EF=MFesedimentMFebackground

where M/Fe_sediment_ is the content of metals in the sediment, M/Fe_background_ is the content of metals in the average background [48]. The enrichment factors are categorized into 5 classes: depletion of mineral enrichment (EF < 2), moderate enrichment (2 < EF < 5), significant enrichment (5 < EF < 20), very high enrichment (20 < EF < 40), and extremely high enrichment (EF > 40).

#### 2.4.6. Geoaccumulation Index (Igeo)

The geoaccumulation index exhibits the relative accumulation of a heavy metal versus a geochemical background in sediment and is calculated by the following equation [49]:

(5) $$\mathrm{Igeo}=\log_{2}\left[\frac{\mathrm{Cn}}{1.5\times \mathrm{Bn}}\right]$$

Cn represents the concentration of heavy metal n within the sediment matrix, while Bn denotes the geochemical background of the same metal, with the constant value of 1.5 serving as a correction coefficient to account for the variations in background concentrations attributable to lithogenic influences. The upper crust average value was used for calculation since Thailand lacks a geochemical background report McLennan [50]. The pollution levels are categorized into 7 classes as follows: unpolluted (Igeo ≤ 0), unpolluted to moderately polluted (0 < Igeo ≤ 1), moderately polluted (1 < Igeo ≤ 2), moderately to heavily polluted (2 < Igeo ≤ 3), heavily polluted (3 < Igeo ≤ 4), heavily to extremely polluted (4 < Igeo ≤ 5), extremely polluted (5 < Igeo ≤ 6) [51].

### 2.5. Statistical Analysis

The one-way ANOVA test was conducted to analyze the importance of seasonal changes in surface sediment and water data. Furthermore, Pearson’s correlation and Principal Component Analysis (PCA) were employed to investigate the relationships between different variables and to assess the impact of human-caused heavy metal contamination. All statistical analyses were performed using the SPSS version 23.

## 3. Results

### 3.1. Physicochemical Parameters of Surface Sediments

The physicochemical characteristics of surface sediments and aquatic environments, including textural attributes (sand, silt, and clay), pH levels, electrical conductivity (EC), and organic matter (OM) content, are shown in Table 1. The surface sediments under investigation were predominantly clay and silt, with the fine fraction (clay + silt) surpassing 90% in all samples. Silt emerges as the preeminent component with its average content ranging between 64.18% and 79.96%. The sediments are, thus, categorized as silty clay loam from their textural perspectives. The pH is a pivotal indicator for evaluating water quality and contamination in marine and coastal ecosystems. The pH values recorded in this investigation ranged from 4.13 to 7.52. These signify a continuum from acidic to alkaline conditions.

### 3.2. Heavy Metal Concentrations in Surface Sediments and Water of Pattani Bay

Individual concentrations of nine heavy metals in sediments and water during wet and dry seasons are shown in Table 2 and Table 3. Spatial variations in dissolved heavy metal concentrations in water during sampling periods are also given in Table 2. The highest mean heavy metal concentration was observed during the wet season at the Yaring River site. The most elevated levels of lead (Pb) in water samples were also detected during the wet season at the same site.

The average heavy metal concentrations in surface sediments from the two rivers flowing into Pattani Bay of Cd (0.28–0.75 mg/kg), Cr (18.12–24.28 mg/kg), Cu (8.11–27.14 mg/kg), Fe (22,753–32,982 mg/kg), and Pb (23.66–127.06 mg/kg) were higher than those found in surface sediments located within and outside the bay (Table 3). These elevated heavy metal concentrations in the river sediments suggest that human activities along the Pattani and Yaring rivers may contribute to the increases in contamination.

The Pearson’s correlation coefficients among investigated heavy metals in surface sediments were presented in Table 4. The statistical analysis confirmed that physicochemical factors (OM, pH, and EC) were significantly correlated with some heavy metals. The pH was positively correlated with Cu (r^2^ = 0.433, *p* < 0.01), Mn (r^2^ = 0.448, *p* < 0.01), and Pb (r^2^ = 0.494, *p* < 0.01), whereas the EC was positively correlated with Cu (r^2^ = 0.319, *p* < 0.05). The correlation analysis showed positive correlations among most studied heavy metals. However, Fe was not significantly correlated with Cd, Mn, and Pb. The Mn also showed no statistical correlation with Co, Cr, Ni, and Pb.

### 3.3. Assessment of Heavy Metal Pollution in Surface Sediments and Water

#### 3.3.1. Heavy Metal Evaluation Index (HEI)

**Figure 2:**
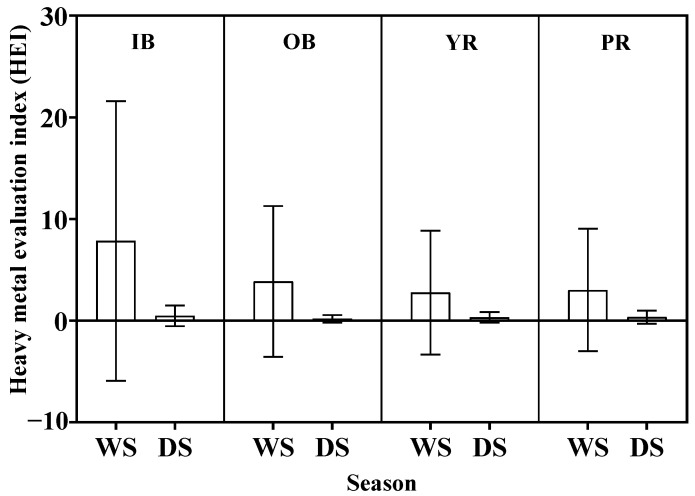
Seasonal variations in heavy metal evaluation index (HEI) in surface water from in-bay (IB), off-bay (OB), Yaring River (YR), and Pattani River (PR) during wet season (WS) and dry season (DS).

The assessments of the heavy metal evaluation index (HEI) for all water samples collected throughout both sampling periods demonstrated low levels of contamination (HEI < 40) (Figure 2). The predominant factors contributing to the elevated contamination levels in Pattani Bay could be due to moderate domestic activities and chemical fertilizer applications.

#### 3.3.2. Contamination Factor (CF) and Pollution Load Index (PLI)

Levels of heavy metal contamination in this study were estimated using contamination factor (CF) values (Table 5). The results revealed that averages of all metal CFs were in the following hierarchical order: Pb > Cd > Co > Mn > Fe > Zn > Cu > Ni > Cr. These indicated that all aforementioned heavy metals have contaminated surface sediments within Pattani Bay. The Cr, Fe, and Ni contamination levels were classified as low. In addition, Co, Cu, Mn, and Zn demonstrated low to moderate contamination levels. The remaining Cd and Pb CF values indicated similar moderate to considerable contamination level. The greatest CF value was 5.37 for Pb at Pattani River during the wet season. Lead contamination across all sampling sites can be attributed to the discharge of Pb originating from historical mining operations. It is also noteworthy that the CF values for Co, Cr, Cu, Fe, Mn, Ni, and Zn across all sampling locations were well below 3. These suggested that overall contamination levels for those elements were relatively low to moderate.

**Figure 3:**
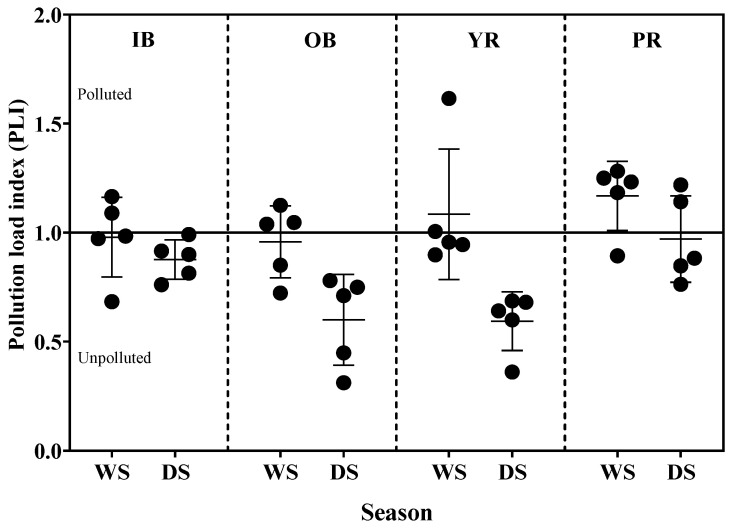
Seasonal variations in Pollution Load Index (PLI) of heavy metals in surface sediments from in-bay (IB), off-bay (OB), Yaring River (YR), and Pattani River (PR) during the wet season (WS) and dry season (DS).

A comparative analysis of heavy metals in surface sediments can also be derived from pollution load index (PLI) data illustrated in Figure 3. The PLI quantifies the multiplicative factor by which the calculated metal concentration in sediment samples that exceeds the background levels. The average PLI values of the analyzed heavy metals fluctuated between 0.79 and 1.17 and 0.59 and 0.97 during the wet and dry seasons, respectively. These values suggest that the pollution of the investigated sediment ranged from unpolluted to polluted as follows: off-Pattani Bay < in-Pattani Bay < Yaring River < Pattani River.

**Figure 4:**
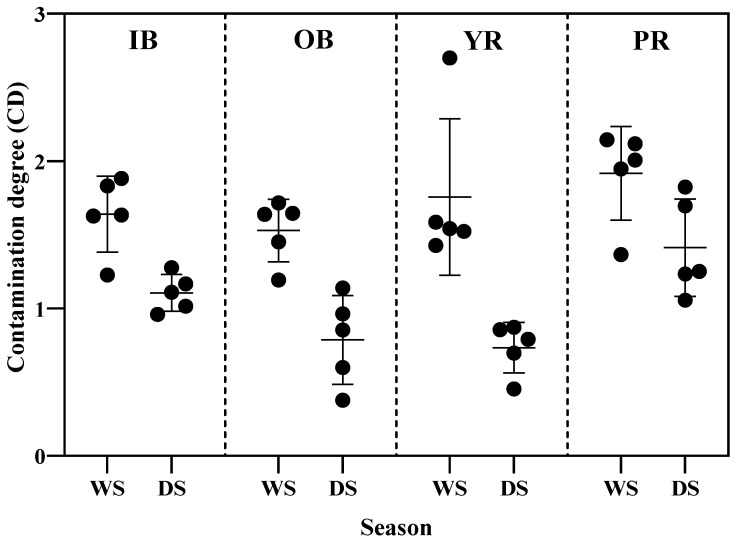
Seasonal variations in Contamination Degree (CD) of heavy metals in surface sediments from in-bay (IB), off-bay (OB), Yaring River (YR), and Pattani River (PR) during the wet season (WS) and dry season (DS).

Additionally, the average contamination degrees (CD) ranged from 1.53 to 1.92 and 0.73 to 1.41 during wet and dry seasons, respectively. They indicated that all heavy metal contents in surface sediments had a low degree of contamination (Figure 4). The CD values of the two rivers were higher than those of the in- and off-Pattani Bay. These confirm that the anthropogenic activities along the river were the source of heavy metal contamination into the bay.

#### 3.3.3. Enrichment Factor (EF) and Geoaccumulation Index (Igeo)

**Figure 5:**
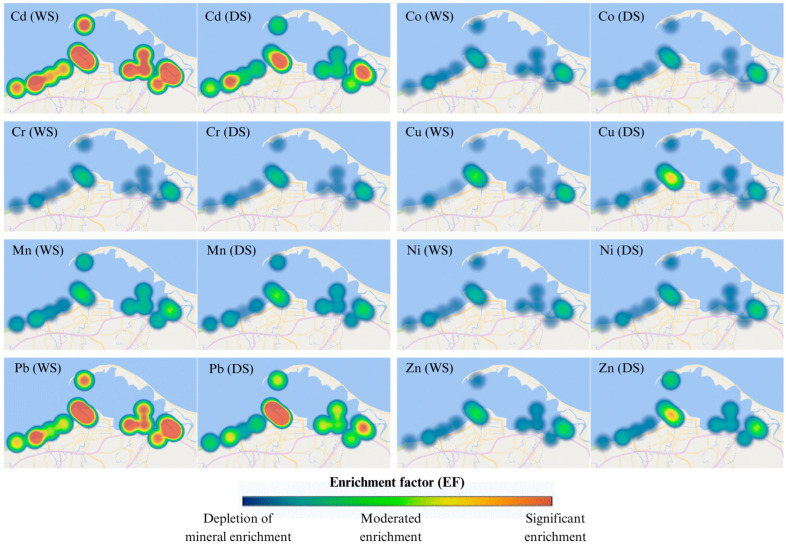
Seasonal variations in enrichment factor (EF) of heavy metals in surface sediment from in-bay, off-bay, Yaring River, and Pattani River during the wet season (WS) and dry season (DS).

**Figure 6:**
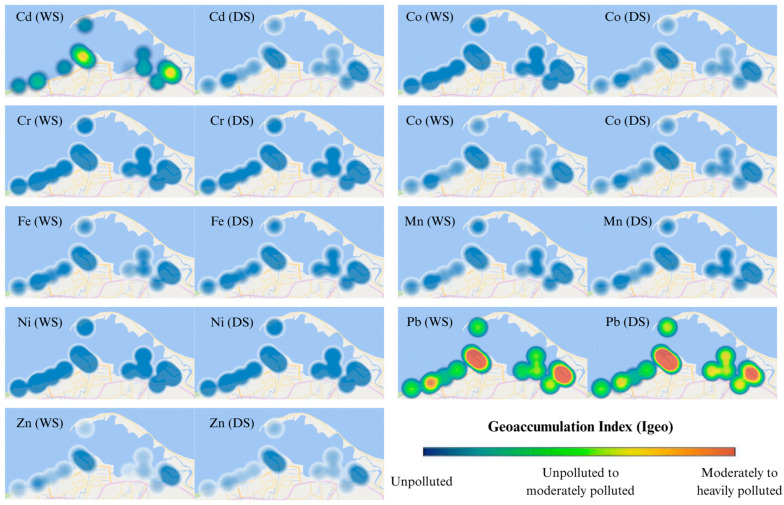
Seasonal variations in geoaccumulation index (Igeo) of heavy metals in surface sediments from in-bay, off-bay, Yaring River, and Pattani River during the wet season (WS) and dry season (DS).

The enrichment factor (EF) and geoaccumulation index (Igeo) values for studied heavy metals were presented in Figure 5 and Figure 6. The EF and Igeo values were determined across different stations from depletion of mineral enrichment to moderated enrichment, and unpolluted to heavily polluted, respectively. The average EF values of Co, Cr, Cu, Mn, Ni, and Zn were lower than 2.0 or 3.0, indicating the depletion of minerals enrichment or moderate enrichment in surface sediments. Whereas the average EF values of Cd and Pb were between 2 and 20, demonstrating that these two metals were moderately enriched to significantly enriched in surface sediments.

The average Igeo values of Co, Cr, Cu, Fe, Mn, Ni, and Zn were lower than 0.0. That indicated unpolluted contamination of these heavy metals in the surface sediments of Pattani Bay (Figure 6). Conversely, Igeo values of Cd and Pb were unpolluted to moderately polluted, and moderately to heavily polluted, respectively. The Igeo value of Pb at Pattani River was the highest (Igeo > 2.00). This demonstrates that this area was moderately to heavily polluted with Pb.

### 3.4. Principal Component Analysis and Source Identification

**Figure 7:**
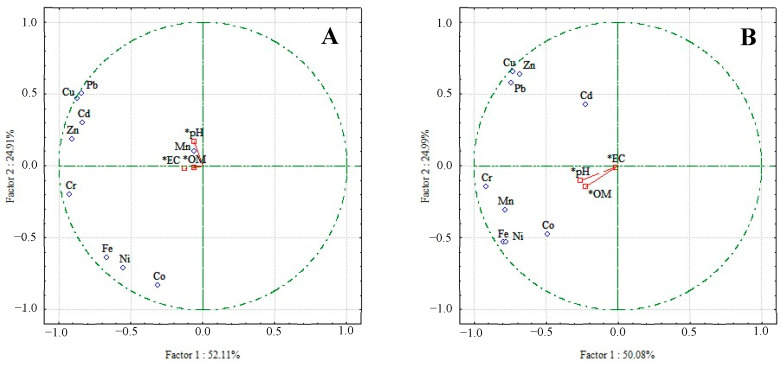
PCA results of heavy metals in surface sediments during the (**A**) wet season and (**B**) dry season; * indicates the supplementary factor.

Previous studies have demonstrated that heavy metals in sediments can come from different natural and anthropogenic sources. Principal component analysis (PCA) was extracted if its cumulative variance exceeded 70% or the eigenvalue exceeded 1 [55,56]. The principal component analysis identified PCs for sediment (Figure 7). River inputs and historical deposition upstream of dams are essential sources of toxic heavy metal sediment contamination [57]. Remobilization between water and the bottom layers of sediments is regulated by the water discharge of the two rivers. The PCA was applied to identify and interpret the metal relationships and discuss similar contamination sources [58]. The PCA output emphasized comparable variance explained by the first two vectors in the wet and dry seasons (PC1 = 52.11% and 50.08%; PC2 = 24.91% and 24.99%), respectively. These results showed that the eigenvalues from three components exceeded the threshold of 1 for the wet season (PC1 = 4.69, PC2 = 2.24, PC3 = 1.19). On the contrary, only two components were >1 (PC1 = 4.51, PC2 = 2.25) for the dry season. The differences in dominant origins were observed for the investigated heavy metals in both seasons, except Mn. Possible similar sources for Cd, Cu, Pb, and Zn were expected. The same evidence also occurred for Co, Cr, Fe, and Ni. The current heavy metal pollution in Pattani Bay, therefore, is quite serious according to these current results. The heavy metal contamination in surface sediments, especially Cd and Pb, can lead to high ecological health risks.

## 4. Discussion

Heavy metals tend to remain suspended in the water column rather than being deposited in benthic sediments in the presence of robust water currents [59]. The predominant factor influencing and resulting in elevated metal concentrations is the substantial proportion of fine-grain sediments [60]. Fine sediments can adsorb heavy metals from aquatic environments and demonstrate a considerable ability to sequester these metals [61]. Fine-grain sediments are the primary determinant of heavy metal concentrations in the marine ecosystem. Fine sediments exhibit greater propensity to absorb heavy metals from water compared to their coarser counterparts in general and also possess significant capabilities (such as surface area, cation exchange capacity, and adsorption) to retain heavy metals [60,61,62,63,64].

The significant temporal variations in heavy metal concentrations are probably due to seasonal fluctuations. These considerable differences in heavy metal concentrations are unusual during this short sampling period. This study showed different sediment parameters between wet and dry seasons (Table 1). Most pH and OM values in the wet season were higher than in the dry season. However, several studies indicated that chemical properties of heavy metals in water and sediments are associated with other environmental factors, including atmospheric deposition, high dynamics of marine water, tidal and seasonal currents, and changes in pollution load of anthropogenic sources. These can cause temporal variations in mobility, bioavailability, and enrichment of heavy metals during a short time. The ability of aquatic sediments to adsorb heavy metals is related to their organic matter content [31]. The organic matter in sediments acts as a significant sink for heavy metals. The functional groups present in organic matter, such as carboxyl, hydroxyl, and sulfhydryl groups can form complexes with heavy metals and, thus, reduce their bioavailability [65].

A study indicated that pollutant sources along the Pattani and Yaring Rivers, including untreated wastewater, municipal effluents, as well as industrial discharges, have been continuously and directly released into these rivers [60]. The effluents from the Yaring and Pattani Rivers can be considerable pathways for toxicant contamination in Pattani Bay. The problem of Pb contamination in water and sediment in Pattani River has existed for a long period of time and continues due to lack of actions to solve this problem from responsible governmental or nongovernmental authorities [12]. The heavy metals present in the water and surface sediments of Pattani Bay have propensity to exchange readily with the contaminated effluents from the Pattani and Yaring Rivers. These are attributed to weak water currents of the waterways. There is, thus, sufficient temporal opportunity for heavy metals to be adsorbed by suspended particulate matters. These could eventually lead to their deposition onto surface sediments.

A comparative analysis of heavy metal concentrations across dry and wet seasons revealed that certain heavy metals had elevated concentrations during the wet season relative to the dry season. This phenomenon can be attributed to precipitation that results in excessive leaching of surface sediments, thereby diluting metal concentrations and facilitating increased mobility of metals within the sediments [66,67,68]. The concentrations of specific metals, notably cadmium (Cd) and lead (Pb), were among those found to be elevated during the wet season. These were likely due to the runoff of pollutant-containing effluents from the adjacent river basins. Particularly in the Pattani Bay region, all investigated heavy metals demonstrated a consistent pattern in which the concentrations of heavy metals increased in proximity to the riverine systems. The pollution sources along the Pattani and Yaring rivers, including untreated wastewater, municipal effluents, as well as industrial discharges, have been continuously and directly released into the river and subsequently to the sediments. Numerous studies have indicated that the physicochemical characteristics of metals, water, and sediments may account for the observed temporal variations in the mobility, bioavailability, and enrichment of heavy metals over short time scales. This is because those characteristics are intertwined with various environmental factors such as atmospheric deposition, the dynamic nature of marine waters, tidal and seasonal currents, and fluctuations in pollution loads from anthropogenic sources. The trace metal concentrations in both sediments and water during the rainy season may be lower than those observed in the summer [60]. This outcome can be related to significant disturbances in sediments caused by the heightened wave activity during the rainy season, together with the potential for rainwater to enhance mobility and dilution of trace metals, resulting in concentration reduction in the sediments [67]. Furthermore, the anthropogenic pollutant loads associated with activities such as shipping and fishing during the rainy season tend to be decreased or ceased in certain areas. The input of metals from vessels may decrease following this anthropogenic activity reduction, resulting in lower metal concentrations in the sediment. In contrast, the influx of metals begins to rise again when anthropogenic activities are escalated during the dry season. It could facilitate an increase in metal levels again [69,70].

The elevated levels of Pb could be attributed to the tin mine discharge from the upper basin in Yala province. A previous study supports this by documenting that the Pb contamination in the water and sediment of the Pattani River basin originated from the mine area [12,14]. In addition, boat-repairing activities at the Pattani River mouth were also the source of Pb contamination in Pattani Bay [12]. Iron (Fe) concentration was the highest in the surface sediments, followed by Mn > Pb > Zn > Cr > Ni > Cu > Co > Cd, respectively. The distribution of these metals across sampling locations revealed that some metals exhibited similar patterns. The most concentrations of Co, Cr, Cu, Fe, Mn, Ni, and Zn in this study area were lower when compared to the average background values in a report by McLennan [50]. Conversely, the average concentrations of Cd and Pb were higher than the background concentrations [50], indicating some degree of heavy metal enrichment in the study area. Metal concentrations in surface sediments in this study were either significantly higher or lower than those found in other regions of Thailand (Table 6).

Almost all heavy metals showed significant positive correlations in Pearson’s correlation analysis, suggesting similar pollution sources [71,72]. Manganese showed no significant correlation to all heavy metals. This is likely due to differences in transport mechanisms in the aquatic environments. The highly significant positive correlations between most soil heavy metals indicated the possible comparable source inputs, which could either be natural or anthropogenic [71].

**Table 6:** Comparison of heavy metals in surface sediment data in Thailand and regional studies (unit: mg/kg).

Country	Location	Cd	Co	Cr	Cu	Fe	Mn	Ni	Pb	Zn	Reference
Thailand	In-Pattani Bay	0.43	7.82	21.71	11.68	25,196.00	746.77	14.16	78.42	42.00	This study
Thailand	Off-Pattani bay	0.42	8.01	19.24	8.49	25,075.89	448.14	13.32	54.63	40.09	This study
Thailand	Yaring River	0.43	8.59	20.31	11.40	28,165.17	417.08	14.22	60.94	49.68	This study
Thailand	Pattani River	0.45	7.41	23.88	24.30	26,743.70	465.45	12.92	120.12	69.81	This study
Thailand	Western Gulf of Thailand	Na	10.44	50.78	12.25	Na	Na	25.86	21.35	46.17	Liu, Shi [73]
Thailand	Chao Phraya River	0.58	Na	Na	214	16,636	419	Na	62.6	240	Wijaya, Ohde [74]
China	DYB	0.07		59.03	16.46				37.01	87.81	Zhao, Ye [75]
China	Daya Bay	0.052	12.7	Na	20.8	Na	837	31.2	45.7	113	Gao, Arthur Chen [76]
China	Pearl River Estuary	Na		106	45.7				57.9	176.8	Yu, Yan [77]
China	Beibu Gulf	0.01–0.153	Na	3.07–85.07	1.06–25.23	Na	Na	Na	9.42–43.05	4.59–112.6	Yi, Song [78]
Sediment quality standard	Proposed SQG-Thailand	0.17	-	72	33	-	-	27.5	35.8	95	PCD [79]
	TEL	0.6	-	37.3	35.7	-	-	18	35	123	MacDonald, Dipinto [80]
	ERL	5	-	70	70	-	-	30	35	120	MacDonald, Dipinto [80]
	LEL	0.6	-	16	16	-	-	16	31	120	MacDonald, Dipinto [80]

Na, data not available; Proposed SQG-Thailand, Proposed Sediment Quality Guideline of Thailand; TEL, threshold effect level; ERL, effects range low; LEL, lowest effect level.

All water samples collected throughout both sampling periods demonstrated low levels of contamination (HEI < 40). However, the HEI method has no defined critical limit. The demarcation of a scale in the HEI method becomes too arbitrary and study-specific [25]. It was found that the HEI values in this study were consistent with the indices used in sediment risk analysis (CF, PLI, EF, CD, and Igeo). Two important indices (i) contamination factor (CF) and (ii) pollution load index (PLI) have been widely used to measure the metal pollution in sediment [26,43,81]. The PLI was used to apply for the determination of the level of heavy metal pollution in the particular studied site [82]. Elevated PLI levels exceeding 1 in the Pattani and Yaring rivers indicate the occurrence of metal(loid) pollution. This observation may be attributed to historical mining operations at these locations. This emphasizes the vulnerability to metal contamination induced by anthropogenic activities and natural processes. Most sampling locations showed PLI values below 1, signifying the absence of pollution. The PLI serves as a valuable metric used to inform communities about the sediment quality that can impacts local inhabitants [83]. Studies conducted across diverse coastal environments have documented PLI values ranging from minimal to significantly elevated, reflecting disparate levels of anthropogenic influences and natural inputs [75,84,85,86]. Furthermore, it provides critical information for policymakers regarding the pollution status of the study area. This underscores the general toxicity of heavy metals [87]. Additionally, the PLI offers vital insights into sediment pollution levels for decision-makers’ policy [88].

The enrichment factor (EF) and geoaccumulation index (Igeo) have broadly been applied for the assessment of heavy metal contamination level in sediment samples over time. The EF values of Co, Cr, Cu, Mn, Ni, and Zn indicate the depletion of minerals enrichment to moderate enrichment in surface sediments. This shows that sources of these metals were from crust material or natural weathering. The average EF value of Pb was the highest/higher at all Pattani River stations than at other stations. The results suggested that anthropogenic activities (from old mining areas) might be a significant source of Pb contamination in the area. A previous study reported that industrial activities, including mining are major contributors to heavy metals (Cd, Cu, Pb, and Zn) released into waterway, which flow into the bay [14].

## 5. Conclusions

This investigation examined the physicochemical characteristics and heavy metal contamination within the surface sediments and aquatic environments of Pattani Bay, Thailand, throughout both wet and dry seasons. The prevalence of fine-grained sediments (silt and clay) in all sampling areas exhibited a greater propensity to absorb heavy metals from water. The pH values signify a continuum from acidic to alkaline conditions, temporal fluctuations in physicochemical characteristics, including pH and organic matter. They imply that seasonal variations significantly affect heavy metal concentration in the aquatic sediments.

The assessment of heavy metal concentrations showed high levels of specific metals, particularly Cd and Pb. This indicates a troubling extent of pollution in certain areas. The significantly higher average concentrations of Cd, Cr, Cu, Fe, and Pb in the sediments of the Pattani and Yaring Rivers compared to those within and outside the bay imply that riverine areas contribute to the increases in heavy metal contamination. The noted temporal variations in heavy metal concentrations between wet and dry seasons can be due to various factors, including precipitation-induced leaching and dilution, hydrodynamics alterations, and anthropogenic pollutant loads.

Using ecological pollution assessment indices, including the heavy metal evaluation index (HEI), contamination factor (CF), pollution load index (PLI), contamination degree (CD), enrichment factor (EF), and geoaccumulation index (Igeo) provided comprehensive insight into contamination status. The HEI is a parameter used to evaluate the combined effect of multiple heavy metals on water quality. The CF, PLI, and CD are used to measure the metal pollution in sediment. The EF and Igeo were applied for the assessment of heavy metal contamination level in sediment samples over time. The HEI indicated low levels of contamination. Using CF values, Cr, Fe, and Ni contamination levels were classified as low. In addition, Co, Cu, Mn, and Zn demonstrated low to moderate contamination levels. The remaining Cd and Pb CF values indicated similar moderate to considerable contamination levels. The PLI values suggest that the sediment in the investigated area ranged from unpolluted to polluted as follows: off-Pattani Bay < in-Pattani Bay < Yaring River < Pattani River. The EF and Igeo determined across different stations revealed depletion of mineral enrichment to moderate enrichment, and unpolluted to heavily polluted, respectively.

Principal Component Analysis (PCA) further clarified the underlying correlations among heavy metals, showing potential similar sources for Cd, Cu, Pb, and Zn. The differences in dominant origins were observed for the investigated heavy metals in both seasons, except Mn.

In summary, this research highlights that Pattani Bay is experiencing considerable heavy metal pollution, especially Cd and Pb in surface sediments. Riverine inputs from two rivers serve as heavy metal sources. The findings showed that the heavy metal pollution present is quite serious, particularly concerning Cd and Pb, which could lead to high ecological health risks and is necessary to focus on implementing environmental management strategies. The strategies shall include knowledge management, urban and industrial wastewater management, which could help with reducing heavy metal contamination in Pattani Bay. Both governmental and industrial agencies are important for the mitigation of pollution by restoring and preventing heavy metal contamination into the environment. Their involvements can be improved by drawing community concerns.

## Figures and Tables

**Table 1:** Physiochemical parameters of surface sediments from in-bay (IB), off-bay (OB), Pattani River (PR), and Yaring River (YR) of Pattani Bay during the wet season (WS) and dry season (DS).

Areas	Sand (%)		Clay (%)		Silt (%)		Texture		pH		EC (dS/m)		OM (%)	
	WS	DS	WS	DS	WS	DS	WS	DS	WS	DS	WS	DS	WS	DS
IB	1.78	0.20	26.64	34.72	71.58	64.18	Silty clay loam		7.52	7.42	11.96	14.39	13.00	10.07
OB	6.64	7.04	23.38	23.98	69.98	69.78	Silty clay loam		6.78	6.75	7.12	10.58	10.38	7.44
PR	0.83	1.28	31.46	28.60	67.71	70.12	Silty clay loam		5.44	5.59	4.71	2.65	12.49	9.82
YR	0.06	0.58	25.40	19.46	75.54	79.96	Silty clay loam		4.17	4.13	4.36	6.52	11.51	12.41

EC: electrical conductivity; OM: organic matter.

**Table 2:** Average (mean), minimum (min), and maximum (max) heavy metal concentrations in the surface water (µg/L) from in- and off-Pattani Bay (IB and OB), Yaring River (YR), and Pattani River (RR) during the wet season (WS) and dry season (DS) with water quality standards (mg/L).

Heavy Metal		Average Heavy Metal Concentration (µg/L)								Water Quality Standards (mg/L)		
		IB		OB		YR		PR				
		WS	DS	WS	DS	WS	DS	WS	DS	WHO ^a^	EPA ^b^	EU ^c^
Cd	Mean	3.41 *	0.45	26.69	1.06	5.88 *	1.10	4.56 *	1.90	0.003	0.003	0.003
	Min	0.20	0.30	21.00	0.30	4.20	0.30	2.40	0.60			
	Max	7.90	0.60	39.3	1.70	8.10	2.90	6.10	3.40			
Co	Mean	7.15	14.23 *	136.89 *	13.65	5.90	16.08 *	4.49	16.74 *	-	-	-
	Min	2.00	2.00	98.10	4.00	2.00	2.00	1.00	3.90			
	Max	12.30	23.90	239.00	23.40	13.70	31.90	11.70	31.90			
Cr	Mean	16.38	94.40 *	102.55	Nd	6.87 *	Nd	5.94	40.30 *	0.05	0.05	0.05
	Min	2.50	48.80	78.60	Nd	2.50	Nd	2.40	40.30			
	Max	40.50	175.80	147.00	Nd	14.60	Nd	9.80	40.30			
Cu	Mean	9.16	7.77	37.53 *	4.39	5.28	5.76	3.75	10.85 *	2.00	2.00	2.00
	Min	4.50	3.70	30.40	1.20	1.70	1.20	2.40	2.50			
	Max	13.50	11.00	49.00	7.40	7.90	14.70	9.20	17.10			
Fe	Mean	3.30	6.08 *	132.05 *	9.13	6.23	6.72	3.40	17.38 *	0.30	0.20	-
	Min	1.10	Nd	5.71	2.20	2.20	1.10	0.50	4.30			
	Max	4.40	13.00	70.75	19.80	12.10	19.60	8.80	26.10			
Mn	Mean	Nd	2.50	8.55	3.21	2.30	3.28	0.75	4.31 *	0.40	0.50	-
	Min	Nd	0.50	3.00	0.50	1.50	0.20	0.50	0.50			
	Max	Nd	10.00	14.10	8.00	3.40	10.90	1.00	16.60			
Ni	Mean	13.73	9.16	117.43 *	10.73	9.08	17.97 *	10.75	17.70 *	0.07	0.02	0.02
	Min	4.40	2.30	86.3	Nd	Nd	7.00	5.90	1.20			
	Max	31.70	23.40	188.4	25.80	30.30	39.80	19.50	32.80			
Pb	Mean	184.25 *	8.58	386.55 *	35.82	151.87 *	16.98	170.23 *	15.64	0.01	0.01	0.01
	Min	127.00	4.90	293.00	9.80	87.90	2.50	131.80	2.50			
	Max	251.50	19.60	542.00	58.60	212.40	44.20	297.50	29.50			
Zn	Mean	Nd	12.66	3.77	15.87 *	8.98	11.63 *	9.73	10.19	5.00	-	-
	Min	Nd	9.90	0.70	2.40	7.30	7.90	5.60	5.00			
	Max	Nd	17.80	5.70	31.70	10.70	19.10	13.20	17.10			

Nd: non-detectable, * Statistically significant difference between seasons at *p* < 0.05. ^a^ WHO [52], ^b^ EPA [53], ^c^ EU [54].

**Table 3:** Average (mean), minimum (min), and maximum (max) heavy metal concentrations in the surface sediments (mg/kg) from in- and off-Pattani Bay (IB and OB), Yaring River (YR), and Pattani River (RR) during the wet season (WS) and dry season (DS) with background concentration of each heavy metal in the Earth’s crust (average shale) (mg/kg).

Heavy Metal		Average Heavy Metal Concentration (mg/kg)								Background (Upper Crust) ^a^
		IB		OB		YR		PR		
		WS	DS	WS	DS	WS	DS	WS	DS	
Cd	Mean	0.58	Nd	0.61	Nd	0.67	0.75	0.67 *	0.28	0.098
	Min	0.41	Nd	0.41	Nd	0.47	0.07	0.47	0.02	
	Max	0.69	Nd	0.75	Nd	1.00	0.07	0.83	0.47	
Co	Mean	9.36 *	6.67	8.23	7.41	9.52 *	7.66	8.03 *	6.80	17
	Min	7.61	3.51	5.64	5.82	9.12	5.45	4.74	5.33	
	Max	10.93	8.39	9.77	8.52	10.23	10.13	8.99	8.88	
Cr	Mean	21.38 *	17.10	18.61	24.80 *	22.49 *	18.12	24.48	23.27	83
	Min	14.72	5.54	11.92	19.06	19.99	10.69	21.02	18.68	
	Max	25.80	23.52	24.19	28.86	27.82	21.93	30.73	27.21	
Cu	Mean	8.49	8.49	9.31	14.04 *	14.69	8.11	21.47	27.14	25
	Min	3.45	4.08	5.70	10.67	6.98	4.10	8.64	18.71	
	Max	11.50	13.61	12.34	17.18	41.50	9.72	28.67	39.89	
Fe	Mean	28,904.25 *	21,247.53	23,642.00	26,750.01 *	32,982.84 *	23,347.50	30,734.40 *	22,753.00	35,200
	Min	21,767.87	11,679.66	15,576.53	20,692.12	30,842.11	14,979.10	25,482.41	18,408.15	
	Max	35,792.95	26,382.29	28,565.03	32,266.44	35,886.52	26,733.37	34,360.85	26,740.99	
Mn	Mean	555.09 *	341.19	845.53 *	648.02	534.07 *	300.13	506.00	424.89	600
	Min	412.17	155.69	510.68	476.33	396.18	137.28	374.09	340.10	
	Max	716.13	555.66	1005.49	871.16	696.77	495.82	666.67	593.27	
Ni	Mean	17.17 *	9.48	14.82	13.49	17.64 *	10.82	15.46 *	10.39	44
	Min	12.56	4.52	9.77	9.36	16.30	7.65	13.55	7.38	
	Max	19.96	12.35	17.70	15.91	18.95	12.97	16.92	12.06	
Pb	Mean	80.45 *	28.81	95.13 *	61.72	98.22 *	23.66	127.06	113.18	17
	Min	56.14	10.95	71.68	51.41	66.68	8.26	68.99	80.12	
	Max	98.86	45.40	114.78	69.40	182.83	30.08	173.85	138.44	
Zn	Mean	42.92 *	37.25	36.52	47.49 *	53.65	45.72	59.63	79.99 *	71
	Min	31.78	21.49	25.58	41.84	38.87	30.44	50.13	55.62	
	Max	51.56	45.79	46.70	53.63	113.12	52.58	69.06	140.12	

Nd: non-detectable, * Statistically significant difference between seasons at *p* < 0.05. ^a^ McLennan [50].

**Table 4:** Pearson correlation coefficient matrices among heavy metal levels, organic matter (OM), pH and electrical conductivity (EC) in sediment samples collected from Pattani Bay, Yaring River, and Pattani River (*n* = 60).

	OM	pH	EC	Cd	Co	Cr	Cu	Fe	Mn	Ni	Pb	Zn
**OM**	1											
**pH**	−0.157	1										
**EC**	0.277	0.659 **	1									
**Cd**	0.096	0.193	0.223	1								
**Co**	0.122	−0.126	0.048	0.323 *	1							
**Cr**	0.055	0.117	0.254	0.500 **	0.867 **	1						
**Cu**	0.091	0.433 **	0.319 *	0.698 *	0.541 **	0.784 **	1					
**Fe**	0.099	−0.223	0.031	0.288	0.900 **	0.863 **	0.494 **	1				
**Mn**	0.298	0.448 **	0.251	0.396 *	0.069	0.189	0.570 **	−0.127	1			
**Ni**	−0.042	0.045	0.162	0.470 **	0.847 **	0.937 **	0.687 **	0.874 **	0.35	1		
**Pb**	0.175	0.494 **	0.279	0.606 **	0.318 *	0.533 **	0.887 **	0.181	0.825 **	0.378 *	1	
**Zn**	0.098	0.034	0.159	0.325 *	0.912 **	0.849 **	0.587 **	0.812 **	0.029	0.819 **	0.361 *	1

* *p* < 0.05 (2—tailed); ** *p* < 0.01 (2—tailed).

**Table 5:** Contamination factors (CFs) for surface sediment from in- and off-Pattani Bay (IB and OB), Yaring River (YR), and Pattani River (RR) during wet season (WS) and dry season (DS).

Heavy Metal	Contamination Factor (CF) *							
	IB		OB		YR		PR	
	WS	DS	WS	DS	WS	DS	WS	DS
Cd	4.68	1.89	4.49	1.94	5.12	1.45	5.19	1.76
Co	1.48	1.33	1.68	1.20	1.71	1.37	1.44	1.22
Cr	0.24	0.32	0.27	0.22	0.29	0.23	0.31	0.30
Cu	0.37	0.56	0.34	0.34	0.59	0.32	0.86	1.09
Fe	0.68	0.76	0.83	0.61	0.94	0.67	0.65	0.79
Mn	1.31	1.01	0.86	0.53	0.83	0.47	0.79	0.66
Ni	0.50	0.46	0.58	0.32	0.60	0.37	0.53	0.35
Pb	4.02	2.61	3.40	1.22	4.15	1.00	5.37	4.78
Zn	0.52	0.68	0.61	0.53	0.77	0.65	0.85	1.14

* CF < 1 = low contamination; 1 ≤ CF < 3 = moderate contamination; 3 ≤ CF < 6 = considerable contamination; CF ≥ 6 = very high contamination.

## Data Availability

The original contributions presented in the study are included in the article; further inquiries can be directed to the corresponding author.
